# COVID-19 and Inflammatory Bowel Diseases: Risk Assessment, Shared Molecular Pathways, and Therapeutic Challenges

**DOI:** 10.1155/2020/1918035

**Published:** 2020-07-10

**Authors:** Iolanda Valentina Popa, Mircea Diculescu, Cătălina Mihai, Cristina Cijevschi-Prelipcean, Alexandru Burlacu

**Affiliations:** ^1^Institute of Gastroenterology and Hepatology Iași, Romania and ‘'Grigore T. Popa'', University of Medicine, Iasi, Romania; ^2^Department of Gastroenterology Fundeni Clinical Institute, Bucharest, Romania and ‘Carol Davila', University of Medicine and Pharmacy, Bucharest, Romania; ^3^Department of Interventional Cardiology-Cardiovascular Diseases Institute and ‘Grigore T. Popa', University of Medicine, Iasi, Romania

## Abstract

**Background:**

The novel coronavirus SARS-CoV-2 causing COVID-19 disease is yielding a global outbreak with severe threats to public health. In this paper, we aimed at reviewing the current knowledge about COVID-19 infectious risk status in inflammatory bowel disease (IBD) patients requiring immunosuppressive medication. We also focused on several molecular insights that could explain why IBD patients appear not to have higher risks of infection and worse outcomes in COVID-19 than the general population in an attempt to provide scientific support for safer decisions in IBD patient care.

**Methods:**

PubMed electronic database was interrogated for relevant articles involving data about common molecular pathways and shared treatment strategies between SARS-CoV-2, SARS-CoV-1, MERS-CoV, and inflammatory bowel diseases. Besides, Neural Covidex, an artificial intelligence tool, was used to answer queries about pathogenic coronaviruses and possible IBD interactions using the COVID-19 Open Research Dataset (CORD-19). *Discussions*. Few molecular and therapeutic interactions between IBD and pathogenic coronaviruses were explored. First, we showed how the activity of soluble angiotensin-converting enzyme 2, CD209L other receptors, and phosphorylated *α* subunit of eukaryotic translation initiation factor 2 might exert protective impact in IBD in case of coronavirus infection. Second, IBD medication was discussed in the context of possible beneficial effects on COVID-19 pathogeny, including “cytokine storm” prevention and treatment, immunomodulation, interferon signaling blocking, and viral endocytosis inhibition.

**Conclusions:**

Using the current understanding of SARS-CoV-2 as well as other pathogenic coronaviruses immunopathology, we showed why IBD patients should not be considered at an increased risk of infection or more severe outcomes. Whether our findings are entirely applicable to the pathogenesis, disease susceptibility, and treatment management of SARS-CoV-2 infection in IBD must be further explored.

## 1. Introduction

The emergence of novel coronavirus SARS-CoV-2 that causes the COVID-19 disease is yielding a global outbreak with severe threats to public health due to a very high transmissibility rate, being spread in 213 countries with 1,569,504 confirmed COVID-19 cases and 95,269 confirmed deaths as of April 11^th^, 2020 [1] and due to potentially severe complications in elderly or comorbid patients [2].

Given the high infectivity, the complexity of immune mechanisms involved in both SARS-CoV-2 infections as well as in inflammatory bowel diseases (IBD), the immunosuppressive therapy [3] and the gastrointestinal events reported in numerous COVID-19 patients [4], the problem of particular evolution and treatment management in infected IBD patients is of real concern. Although IBD causes are not known, autoimmunity and immune-mediated mechanisms play an essential role in disease pathogenesis [3], and immunosuppressive and immunomodulating drugs are successfully used in IBD therapy [5].

The main questions to answer are whether IBD patients are at an increased risk of SARS-CoV-2 infection in their immunosuppressive or immunomodulating treatment? Or do they have a higher risk of severe clinical course considering that their IBD diagnosis is a comorbidity? Should they stop or change current treatment knowing that this might expose them to develop an IBD flare, as shown in a recent study where 107 IBD patients discontinued their medication during COVID-19 pandemic, and 61 of them required hospitalization for disease aggravation [6]? Might the two diseases share common molecular pathways that could influence each other's evolution? Does IBD medication target COVID-19 pathways? How are current COVID-19 experimental therapies influence IBD course and relapses?

In this paper, we aimed at reviewing the current knowledge about COVID-19 infectious risk status in IBD patients and to describe several molecular insights that could explain why IBD patients appear not to have higher risks of infection and worse outcomes in COVID-19 than the general population. We also attempted to provide scientific support to current data for better, safer, and more precise decisions in IBD patient care in an epidemiological context.

## 2. Methods

The electronic database of PubMed was systematically searched for relevant articles from the inception until June 2020. The search terms used were [“*SARS-CoV-2*” or “*COVID-19*” or “*SARS*” or “*SARS-CoV*” or “*SARS-CoV-1*” or “*MERS-CoV*”] and [“*inflammatory bowel diseases*” or “*ulcerative colitis*” or “*Crohn*”] and [“*molecular pathways*” or “*immunosuppressive*”]. The study selection process included article identification, removing the duplicates, screening titles and abstracts, and assessing eligibility of the selected full texts. Additionally, reference lists of valid articles were checked for studies of relevance. Articles were included if they involved data about common molecular pathways and shared treatment strategies between SARS-CoV-2, SARS-CoV-1, MERS-CoV, and IBD. Journal articles published with full text or abstracts in English were eligible for inclusion.

Additionally, the search engine https://covidex.ai made by the University of Waterloo and New York University was interrogated. Neural Covidex uses natural language processing, state-of-the-art neural network models, and artificial intelligence techniques to answer queries about pathogenic coronaviruses using the COVID-19 Open Research Dataset (CORD-19). CORD-19 is the current largest open dataset available with over 47000 scholarly articles, including over 36000 with full text about COVID-19, SARS-CoV-2, and other coronaviruses from the following sources: PubMed's PMC open access corpus, a corpus maintained by the WHO, bioRxiv and medRxiv preprints. The CORD-19 dataset is available at https://pages.semanticscholar.org/coronavirus-research.

Neural Covidex was interrogated with the following queries: “SARS-CoV-2 and inflammatory bowel diseases,” “COVID-19 and inflammatory bowel diseases,” “SARS and inflammatory bowel diseases,” and “MERS-CoV and inflammatory bowel diseases.” The first interrogation, “SARS-CoV-2 and inflammatory bowel diseases,” returned 40 results. The second query resulted in 25 articles. The third interrogation returned 39 articles, and the fourth query lead to 31 results. After removing the duplicates and assessing the relevance of the research subject, 59 articles were included.

**Figure 1:**
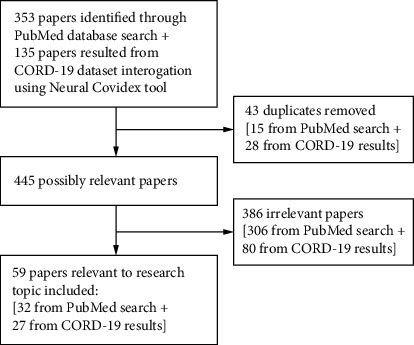
Study selection process and number of papers included.

The study selection process and number of papers identified in each phase are illustrated in the Flowchart (Figure 1).

## 3. Discussions

### 3.1. Why Are IBD Patients Expected to Be More Vulnerable than the General Population Facing the COVID-19 Threat?

It has been shown that IBD patients are at increased risk of pneumonia [7], influenza infection [8], and other infectious complications compared to non-IBD population [9].

Current medication used in IBD consists of anti-inflammatory drugs (mesalazine, corticosteroids), immunosuppressives (Azathioprine, 6-mercaptopurine, Methotrexate), and biologics (Infliximab and Adalimumab—antitumor necrosis factor antibodies, Vedolizumab—monoclonal antibody with gut selectivity that inhibits *α*_4_*β*_7_ integrin, Ustekinumab—IL-12 and IL-23 antagonist, Tocilizumab—anti-IL-6R antibody, Tofacitinib—Janus kinase inhibitor) [10]. IBD patients treated with azathioprine/6-mercaptopurine or immunosuppressant combination therapy and patients older than 50 have been shown to present an increased risk of opportunistic infections [11, 12]. A meta-analysis of randomized control trials comparing antitumor necrosis factor (TNF) therapy with placebo found that anti-TNF therapy significantly increases the risk of opportunistic infections in IBD patients [13, 14]. Vedolizumab has been linked to respiratory and bowel infections, although to a lesser extent than anti-TNF medication [15].

Moreover, recent studies described concomitant digestive symptoms (particularly diarrhea and abdominal pain) associated with COVID-19 [4, 16]. Also, there has been a case report about SARS-CoV-2 gastrointestinal infection causing acute hemorrhagic colitis with colonic injury confirmed endoscopically for which other etiologies have been excluded [17].

Also, preexisting digestive diseases like hepatitis B infection and liver injury are more prevalent in severe COVID-19 cases than in mild ones [2]. Coronaviruses bind to their target cells through angiotensin-converting enzyme 2 (ACE2). Particularly high expressions of ACE2 can be found in ileum, colon, and other digestive tract segments, testis, renal, cardiovascular, and lung tissues [18]. Notably, studies reveal that the inflamed gut in IBD patients is associated with higher ACE2 expression [19], which may facilitate viral entrance.

Finally, coronavirus spike protein binds to the ACE2 receptor and is the substrate for a trypsin-like proteinase found on the host cell surface. Trypsin-like proteinase activates the viral spike protein, which initiates the fusion process between the virus and the host cell. It has been shown that several fecal serine proteinases (including the trypsin-like protease) have increased the activity in IBD [20, 21].

### 3.2. Real-World-Data and Current Experts' Opinions in IBD and COVID-19

Even though the expected consequences of IBD patient's infection with the novel coronavirus are based on solid reasoning, reality does not seem to confirm anticipated poor outcomes.

Currently, the only existing patient data are synthesized in SECURE-IBD, an international IBD reporting database, comprising of 457 patients infected with SARS-CoV-2 (as of April 11^th^, 2020, available at http://covidibd.org/). This database offers information about the number of cases by country and outcomes by patient characteristics (age, sex, diagnosis, disease activity, comorbidities, and medication). Current data does not reveal higher risks of infection, more severe outcomes, or significant differences in disease course between IBD patients and non-IBD patients infected with SARS-CoV-2.

According to SECURE-IBD database, 30% of patients with IBD and SARS-CoV-2 required hospitalization, 7% had a severe disease course either requiring intensive care, invasive ventilation or died, the death rate being 3% (Brenner EJ, Ungaro RC, Colombel JF, Kappelman MD. SECURE-IBD Database Public Data Update. http://covidibd.org/. Accessed on 04/11/20, https://covidibd.org/current-data/).

As the American College of Gastroenterology (ACG) states, currently available information does not attest higher risk of SARS-CoV-2 infection or COVID-19 development for IBD patients, whether or not they are under treatment [22]. At the 2020 meeting of the International Organization for the Study of Inflammatory Bowel Diseases, members and selected content experts voted the appropriateness of multiple statements related to COVID-19 risks. Thereby, the current reasoning was that the risk of infection with SARS-CoV-2 was the same whether a patient had IBD or did not have IBD and that patients with IBD who had COVID-19 did not have a higher mortality compared to patients without IBD [23].

Several studies conducted so far assessed the risks of IBD patients in the context of the COVID-19 pandemic. A population-based retrospective cohort study comparing 232 IBD patients with COVID-19 and non-IBD infected patients found a similar risk of COVID-19 severity (with no difference between patients receiving immune-mediated therapy in the preceding one year and patients without immune-mediated therapy) [24].

No additional COVID-19 risk was also observed for IBD patients in an observational study conducted in Bergamo, Italy (a region with a very high prevalence of infection) [25]. A case series study of 12 IBD patients with confirmed COVID-19 showed no increased risk and associated mortality than the general population, using age-adjusted cumulative incidences [26]. A study on 40 IBD infected patients from Basque Country (Spain), with a third of them being on immunomodulatory therapy, described an overall good prognosis, with no admissions to the intensive care unit [27]. Finally, 15 SARS-CoV-2-positive IBD patients from Nancy and Milan cohorts, treated with biological therapy and/or immunosuppressive drugs, did not report any deaths or intensive care admissions [28].

In conclusion, divergent results were presented by various recent studies: either disease activity was a risk factor for COVID-19 [29], or it was not linked to a higher risk in the SECURE-IBD registry or other studies [28, 30, 31].

### 3.3. IBD Patients Share a Similar Risk of SARS-CoV-2 Infection as the General Population: Possible Counterintuitive Arguments

**Figure 2:**
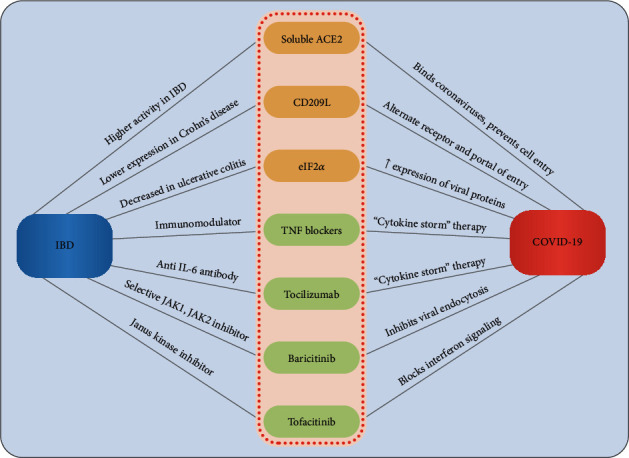
Known and possible interactions between IBD and SARS-CoV-2 immunopathology.

Known and possible interactions between IBD and SARS-CoV-2 immunopathology concerning specific molecular pathways and treatment dynamics are summarized in Figure 2.

#### 3.3.1. Molecular Insights

Although SARS-CoV-2 binds to the cells through ACE2 receptor that appears to have higher expression in IBD, one study found that ACE2 expression in the colonocytes was positively associated with genes regulating viral infection, innate, and cellular immunity, but was negatively associated with viral transcription, protein translation, humoral immunity, phagocytosis, and complement activation [32]. Besides surface-bound ACE2, there is another form of ACE2 that circulates freely in the bloodstream [20]. It has been shown that soluble ACE2 can bind coronaviruses, competing with the surface-bound ACE2, thus preventing viral particles from binding to the cell [33]. Studies suggest that the activity of soluble ACE2 is higher in IBD [34]. This might contribute as a protective factor for IBD patients.

*CD209L* encoded by *CLEC4M* is a dendritic cell-specific ICAM-3-grabbing nonintegrin-related protein that has been described as an alternate receptor and portal of entry for severe acute respiratory syndrome coronavirus (SARS-CoV) [35]. Data showed that CD209L could mediate infection by SARS-CoV, although to a lesser extent than ACE2. CD209L is expressed on type II alveolar cells in human lung [35], but also in the intestine [36], both of which are primary targets for coronavirus infection [37]. Ileal *CLEC4M* that encodes *CD209L* was revealed to have significantly decreased expression in Crohn's disease (CD) [36], suggesting possible protective implications against SARS-CoV-2 in CD patients.

Another molecular interaction between coronaviruses and IBD may be regarding endoplasmic reticulum (ER) stress. Pathogens, as well as autoimmune and inflammatory diseases, induce ER stress, activating several signaling pathways [38]. The phosphorylation of the *α* subunit of eukaryotic translation initiation factor 2 (eIF2*α*) in response to ER stress stimulates viral proteins [39]. A fourfold elevation of the phosphorylated eIF2*α* was found in SARS-CoV infected cells [40]. On the contrary, eIF2*α* has an essential role in mucosal homeostasis [41], and it has been reported that mechanisms induced by ER stress block phosphorylation of eIF2*α* in patients with ulcerative colitis [42].

#### 3.3.2. Therapy Challenges

It is uncertain whether SARS-CoV-2 induces severe forms of COVID-19 due to local viral replication or to subsequent immune system response [43]. The excessive and lengthened cytokine and chemokine response, known as “*cytokine storm*,” leads to high morbidity and mortality due to immunopathologic mechanisms. TNF plays pivotal roles in the “*cytokine storm*” immunopathology. TNF released by monocyte-macrophages boosts the apoptosis of lung epithelial and endothelial cells resulting in vascular leakage and alveolar edema that leads to hypoxia [44]. Also, TNF-mediated T cell apoptosis results in exuberant inflammatory reaction since T cells play a significant role in subsiding hyperactive innate immune responses [45].

Additionally, coronavirus-specific T cells have a crucial role in virus clearance [46]. In a study where TNF was the only neutralized proinflammatory cytokine, mice were protected from SARS-CoV-induced morbidity and mortality [47]. Notably, recent studies showed that anti-TNF was not associated with a significant increased risk of COVID-19 [48] or with more severe outcomes [49]. Thus, anti-TNF therapies in IBD might prove beneficial for patients in the context of COVID-19.

Another key cytokine produced in excess by activated macrophages is IL-6. A potential therapy targeting the host immune system in COVID-19 might be the cytokine blockade of IL-6 [50]. Tocilizumab, an anti-IL-6R antibody used in IBD treatment, is currently tested against COVID-19 in multiple clinical trials (NCT04317092 and NCT04346355).

One of the new drugs that are in clinical development for IBD, but already approved for the treatment of rheumatoid arthritis, is Baricitinib, a selective Janus kinase (JAK1, JAK2) and AP2-associated protein kinase 1 (AAK1) inhibitor. Baricitinib acts as an anti-inflammatory drug through the inhibition of JAK1 and JAK2 and as an antiviral drug inhibiting receptor-mediated endocytosis by blocking AAK1 [23, 51, 52]. Currently, there are ongoing clinical trials for testing Baricitinib as a therapy for COVID-19 (NCT04346147).

Vedolizumab studies show that the rate of serious opportunistic infections and the frequency of tuberculosis were low, and no hepatitis B/C viral reactivation was reported [23, 53]. Studies showed similar opportunistic or viral infections in Ustekinumab therapy compared to placebo [23, 54]. Blocking interferon signaling protected mice from lethal SARS-CoV infection and interferon secretion inhibition by Tofacitinib, as shown in some studies [47], Tofacitinib used in IBD may be protective also against COVID-19.

Mesalazine (an anti-inflammatory drug) and mercaptopurine (a thiopurine) used in IBD therapy were identified as putative repurposable drugs for potential treatment of SARS-CoV-2 through genomics and proteomics analysis using bioinformatics tools [55]. Indeed, studies have shown that thiopurines were able to inhibit in vitro the papain-like protease of SARS-CoV and Middle East respiratory syndrome coronavirus that represents an essential antiviral target essential in viral maturation and the antagonism of interferon stimulation [56, 57]. Additionally, a nationwide cohort study found that thiopurines were not associated with an increased risk of developing COVID-19 [48]. However, care must be taken in evaluating mesalazine as a potential drug for COVID-19 since clinical studies showed possible pulmonary toxicities associations [58].

Finally, although the use of corticosteroids in IBD was identified with a higher risk of severe COVID-19 [24, 30, 48], one study showed that treatment with methylprednisolone decreased the risk of death among patients with ARDS [59]. The use of steroids in COVID-19 remains controversial and available data must be interpreted with caution.

## 4. Conclusions

Specific insights into common molecular and therapy pathways regarding IBD patients infected with SARS-CoV-2 and other pathogenic coronaviruses were described. Physicians need to make clinical decisions concerning the most suitable treatment management in IBD patients requiring immunosuppressive medication. The responsibility lies in correctly balancing infection risk with the risk of IBD relapse in treatment adjustment or discontinuation.

Using the current understanding of SARS-CoV-2 as well as other pathogenic coronaviruses immunopathology, we showed why IBD patients might not be at an increased risk of infection or more severe outcomes. However, COVID-19 is a novel disease with possible different mechanisms of action as other related pathogens, with research still ongoing. Whether our findings are entirely applicable to the pathogenesis, disease susceptibility, and treatment management of SARS-CoV-2 infection in IBD must be further explored.
